# Host gene expression profiling in influenza A virus-infected lung epithelial (A549) cells: a comparative analysis between highly pathogenic and modified H5N1 viruses

**DOI:** 10.1186/1743-422X-7-219

**Published:** 2010-09-09

**Authors:** Alok K Chakrabarti, Veena C Vipat, Sanjay Mukherjee, Rashmi Singh, Shailesh D Pawar, Akhilesh C Mishra

**Affiliations:** 1 Microbial Containment Complex, National Institute of Virology, Sus Road, Pashan, Pune - 411021 India

## Abstract

**Background:**

To understand the molecular mechanism of host responses to highly pathogenic avian influenza virus infection and to get an insight into the means through which virus overcomes host defense mechanism, we studied global gene expression response of human lung carcinoma cells (A549) at early and late stages of infection with highly pathogenic avian Influenza A (H5N1) virus and compared it with a reverse genetics modified recombinant A (H5N1) vaccine virus using microarray platform.

**Results:**

The response was studied at time points 4, 8, 16 and 24 hours post infection (hpi). Gene ontology analysis revealed that the genes affected by both the viruses were qualitatively similar but quantitatively different. Significant differences were observed in the expression of genes involved in apoptosis and immune responses, specifically at 16 hpi.

**Conclusion:**

We conclude that subtle differences in the ability to induce specific host responses like apoptotic mechanism and immune responses make the highly pathogenic viruses more virulent.

## Background

Outbreaks of avian influenza A (H5N1) virus, a highly pathogenic avian influenza (HPAI), are considered as a public health risk with pandemic potential [1]. Understanding the pathology, transmission, clinical features and treatments has become necessary for the prevention and management of such outbreaks [2,3]. The mechanisms responsible for the virulence of HPAI viruses in humans are not completely understood. Viral factors are necessary for productive infection but are not sufficient to explain the pathogenesis of HPAI infection in humans [4,5].

It is well recognized that host immunological and genetic factors also play an important role in the pathogenesis of H5N1 viruses in humans [5,6]. Recent studies have shown that the high fatality rate of avian influenza virus infections is a consequence of the complex interaction of virus and host immune responses which include overactive inflammatory response in the form of hypercytokinemia (cytokine storm), that is initiated inside the infected cells or tissue in response to virus replication resulting in excessive cellular apoptosis and tissue damage [7-9]. *In vitro*, *in vivo *and clinical studies have suggested that H5N1 viruses are very strong inducers of various cytokines and chemokines [Tumor Necrosis Factor (TNF)-alpha, Interferon (IFN)-gamma, IFN-alpha/beta, Interleukin (IL)-6, IL-1, MIP-1 (Macrophage Inflammatory Protein), MIG (Monokine Induced by IFN-gamma), IP-10 (Interferon-gamma-Inducible Protein), MCP-1 (Monocyte Chemoattractant Protein), RANTES (Regulated on Activation Normal T-cell Expressed and Secreted), IL-8], in both humans and animals [10-12]. However, it has also been reported that preventing cytokine response doesn't prevent H5N1 infection and cell death [13]. Hence, further studies are needed to understand the pathogenesis of H5N1 virus infection.

Alveolar epithelial cells and macrophages are the key targets for H5N1 virus in the lungs [14]. Using infection in human lung carcinoma cells we analyzed early and late host responses at 4, 8, 16 and 24 hours post infection by employing gene expression profiling on a microarray platform. A comparative analysis was thus carried out at different time points post infection between highly pathogenic avian influenza A H5N1 virus (HPAI-H5N1), A/Chicken/India/WB-NIV2664/2008(H5N1) and modified recombinant vaccine virus (RG modified H5N1), A/India/NIV/2006(H5N1)-PR8-IBCDC-RG7.

A/Chicken/India/WB-NIV2664/2008 is a recent strain of H5N1 of clade 2.2 circulating in chicken population in India [15] and A/India/NIV/2006(H5N1)-PR8-IBCDC-RG7 is a reverse genetics modified virus generated from HPAI, A/chicken/India/NIV33487/2006 (H5N1) of the same clade [16]. The objective was to understand the host responses at different stages of virus infection at cellular level, which could provide some insight into the biology of virus-host interaction leading to the explanation that how virus infection modulates host cellular environment in A549 cells.

## Materials and methods

### Viruses

Avian Influenza A (H5N1) virus, A/Chicken/India/WB-NIV2664/2008 (WB-NIV2664) isolated from West Bengal (India) outbreak in 2008 [15] and reverse genetics modified H5N1 vaccine virus A/India/NIV/2006(H5N1)-PR8-IBCDC-RG7 (IBCDC-RG7) were used in this study. The vaccine virus was constructed using modified hemagglutinin (HA) (deleting multiple basic amino acids at the cleavage site of HA) and neuraminidase (NA) of A/Chicken/India/NIV33487/06 (H5N1) in the background of A/PR/8/34 (H1N1) using reverse genetics technology at the Molecular Virology and Vaccine Laboratory, Influenza Division, Centers for Disease Control and Prevention, Atlanta. World Health Organization (WHO) has identified this strain as a H5N1 vaccine virus [16].

### Cell line

Human lung carcinoma (A549) cells were maintained in Dulbecco's modified Eagle's tissue culture medium (Invitrogen Life Technologies, Carlsbad, CA, USA) containing 10% fetal calf serum, 100units/ml penicillin, 100 units/ml streptomycin in tissue culture flasks (Corning, USA) at 37°C in a CO_2 _incubator.

### Virus infection

Monolayers of A549 cells at a concentration of 3 × 10^6 ^cells/ml were infected with the viruses at a multiplicity of infection (MOI) of 3. After 1 hour the inoculum was removed; the cells were washed twice with phosphate buffer saline (PBS) and supplemented with growth media. For each virus four different sets of tissue culture flask were infected and harvested at four different time points. Mock infected cells at each time point served as controls. Infection of H5N1 viruses was performed in BSL-3+ facility.

### Preparation of Total Cellular RNA and microarray Hybridization

Total RNA was extracted from the control and infected cells at 4, 8, 16 and 24 hpi using Trizol reagent (Invitrogen Life Technologies, Carlsbad, CA, USA) and purified by the RNeasy kit (Qiagen, Germany) following standard methodology. Amplification of RNA and indirect labeling of Cy-dye was done by Amino Allyl MessageAmp II aRNA amplification kit (Ambion, Austin, TX, USA) using manufacturer's instruction. One hundred nanograms of RNA from control and infected cells were used for the experiments. The RNA was reverse transcribed and amplified according to the manufacturer's protocol. The purified amino allyl aRNA was labeled with Cy3 and Cy5 (Amersham Biosciences, USA) for control and experimental samples respectively. Purified samples were lyophilized, resuspended in hybridization buffer (Pronto Universal Hybridization kit, Corning, USA) and hybridized on the Discover human chip (Arrayit Corporation, Sunnyvale, CA). Hybridization was carried out in a Hybstation (Genomic Solutions, Ann Arbor, MI) and the conditions used were 55°C for 6 h, 50°C for 6 h, and 42°C for 6 h. Scanning was performed at 5-mm resolutions with the Scan array express (PerkinElmer, Waltham, MI). Grid alignment was done using gene annotation files and raw data were extracted into MS EXCEL.

### Data Analysis

Data was analyzed using GENOWIZ Microarray and Pathway analysis tool (Ocimum Biosolutions, Hyderabad, India). Replicated values for genes were merged and median values of the expression ratios were considered for the dataset (2 slides per time point were used). Empty spots were removed by filtering. Dye bias was dealt with by applying loess normalization. Log transformation (log2) was done to stabilize the variation in dataset and median centering was performed to bring down data distribution of dataset close to zero. In order to detect highly expressed genes, fold change analysis was done. Genes with 1.5 folds up/down-regulation were considered as differentially expressed at a p-value < 0.05, Student's t-test. Functional classification of the genes was performed using gene ontology and pathway analysis.

### Quantitative RT-PCR analysis of host genes using SYBR Green I

The differential expression data was validated by quantitative RT-PCR. One hundred nanograms of total RNA from control and infected A549 cells were used for quantitative RT-PCR analysis. Reaction was performed using the QuantiTect SYBR Green RT-PCR kit (Qiagen, Germany) according to the manufacturer's instructions. Reaction efficiency was calculated by using serial 10-fold dilutions of the housekeeping gene- β-actin and sample genes. Reactions were carried out on an ABI 7300 real-time PCR system (Applied Biosystems, Foster City, CA, USA) and the thermal profile used was Stage 1: 50°C for 30 min; Stage 2: 95°C for 15 min; Stage 3: 94°C for 15 sec, 55°C for 30 sec; and 72°C for 30 sec, repeated for 30 cycles. Melting curve analysis was performed to verify product specificity. Reactions were performed in triplicates. All quantitations (threshold cycle [CT] values) were normalized to that of β-Actin to generate ΔCT, and the difference between the ΔCT value of the sample and that of the reference (uninfected sample) was calculated as ΔΔCT. The relative level of gene expression was expressed as 2^-ΔΔCT^. Primer sequences for the genes of interest were designed using Primer Express 2.0 software (Applied Biosystems, Foster City, CA, USA). The primer sequences used in this study are as follows: *JUN FP *5' TCGACATGGAGTCCCAGGA 3'; *JUN RP *5' GGCGATTCTCTCCAGCTTCC 3'; *STAT1 FP 5' *CCATCCTTTGGTACAACATGC 3'; *STAT1 RP *5'TGCACATGGTGGAGTCAGG 3'; *CXCL10 FP *5' TTCAAGGAGTACCTCTCTCTAG 3'; *CXCL10 RP *5' CTGGATTCAGACATCTCTTCTC 3'; *CCL5 FP *5'TACCATGAAGGTCTCCGC 3'; *CCL5 RP *5'GACAAAGACGACTGCTGG 3'; *β-ACTIN FP *5' CATGAAGTGTGACGTGGACATCC 3'; *β- ACTIN RP *5' GCTGATCCACATCTGGAAGG 3'; *BCL2 FP *5' GATGTCCAGCCAGCTGCACCTG 3'; *BCL2 RP *5' CACAAAGGCATCCCAGCCTCC 3'.

## Results

### Host gene expression response to HPAI-H5N1 (WB-NIV2664) virus infection

The number of differentially expressed genes at different time-points after WB-NIV2664 infection is given in Table 1. Gene ontology analysis revealed that the genes involved in immune responses, translation and apoptosis were mostly up-regulated at all the time-points whereas genes involved in cell cycle and transcription were down-regulated. However, it was found that the number of genes was quantitatively different between the different time-points. A list of significantly up and down regulated genes at different post infection time points has been shown in Table S1 (Additional file 1). The 24 hpi time point showed maximum number of differentially expressed genes.

**Table 1:** Summary of differentially expressed genes in response to infection with HPAI-H5N1 and RG modified H5N1 in A549 cell lines.

Time-points	Genes qualifying the quality criteria in replicated experiments	Differentially expressed genes (+/-1.5folds, p < 0.05)	Up-regulated genes	Down-regulated genes
HPAI-H5N1 (A/Chicken/India/WB-NIV2664/2008)				

4 h	253	40	15	25

8 h	208	24	13	11

16 h	254	101	53	48

24 h	309	111	53	58

RG Modified H5N1 (A/India/NIV/2006(H5N1)-PR8-IBCDC-RG7)				

4 h	262	60	36	24

8 h	212	128	67	61

16 h	237	109	58	51

24 h	305	42	22	20

**Figure 1:**
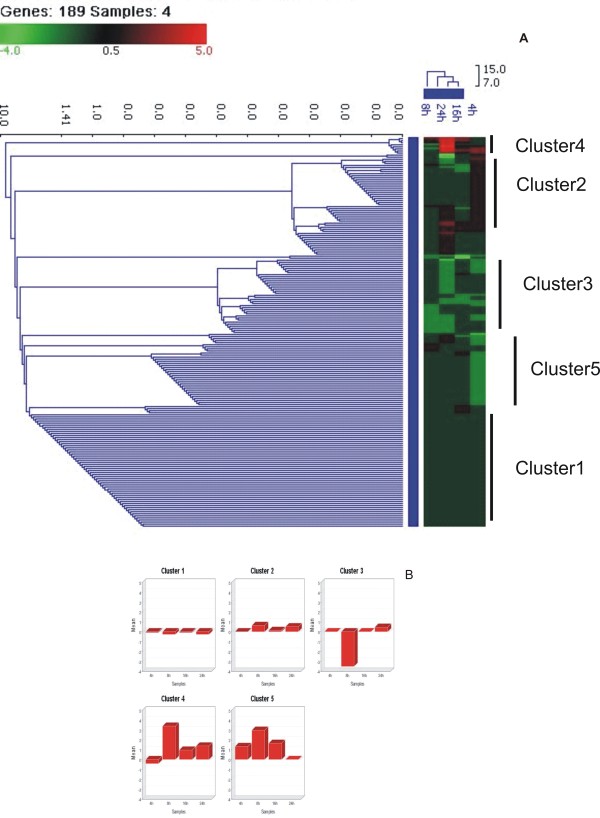
**Hierarchical clustering (A) and k-means clustering (B) of differentially expressed genes of HPAI-H5N1 (A/Chicken/India/WB-NIV2664/2008) infected A549 cells at different post-infection time points**. Expression of genes with p < 0.05 and fold change > +/- 1.5 were considered as differentially expressed. Data presented are averaged gene expression changes for 2 different replicates for each time point.

Cluster analysis of the differentially expressed genes was carried out using GENOWIZ software. K-means clustering and hierarchical clustering methods resulted in identification of 5 distinct patterns of gene expression at different time-points (Figure 1). A total of 189 genes were found common between all the time points. Expression pattern of most of the apoptotic genes was similar and formed a single cluster (Cluster 2 Figure 1). Apoptotic genes BAX, BAK1, TRADD and CASP1 were observed to be significantly up-regulated at 16 and 24 hpi but not at 4 and 8 hpi. Signaling molecules STAT1, IL15RA, GNBP1 were found to be up-regulated at all the time-points and showed a gradual increasing trend from 4 hpi to 24 hpi. Genes coding for ribosomal proteins and IRF1 (Interferon regulatory factor 1) were up-regulated at 4, 8 and 16 hpi but down-regulated at 24 hpi. Genes involved in cell cycle CDK4, CDK5, Cyclin E1, CKN2B, CDKN2D were down-regulated at all time-points post infection. Cytokines IL1, IL2-α and chemokines CXCL10, CCL5 were specifically up-regulated at 16 hpi.

### Host gene expression response to RG modified H5N1 (IBCDC-RG7) virus infection

**Figure 2:**
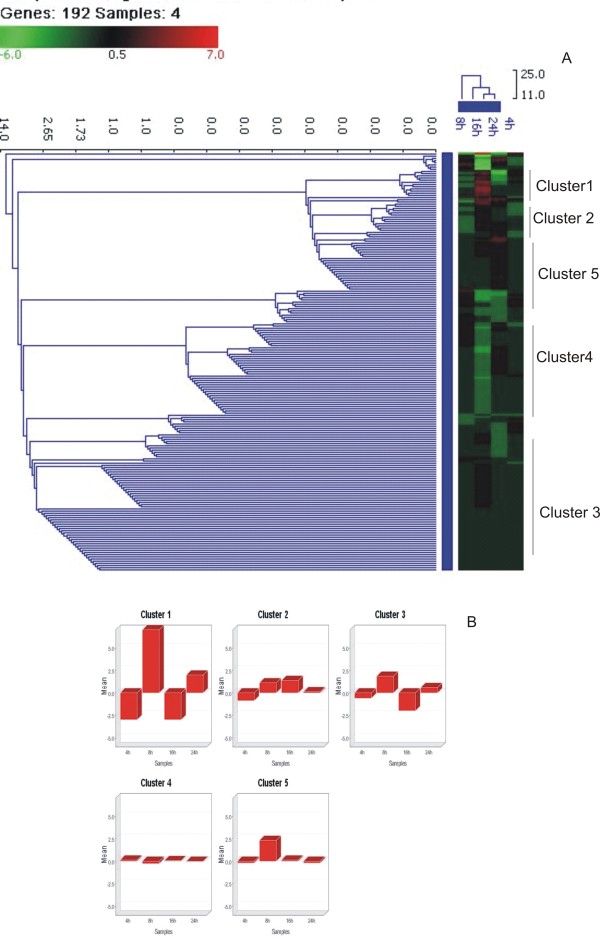
**Hierarchical clustering (A) and k-means clustering (B) of differentially expressed genes of RG modified H5N1 (A/India/NIV/2006(H5N1)-PR8-IBCDC-RG7) infected A549 cells at different post-infection time points**. Expression of genes with p < 0.05 and fold change > +/- 1.5 were considered as differentially expressed. Data presented are averaged gene expression changes for 2 different replicates.

The differentially expressed genes in cells infected with RG modified H5N1 (IBCDC-RG7) virus were involved in similar biological processes (GO analysis) as in response to H5N1 (WB-NIV2664) virus (Table 2). However, there was significant quantitative difference between the expression profiles of the two virus infections. Table S2 (Additional file 2) shows the list of significantly up and down regulated genes at different time point post infection. Contrastingly, genes coding for ribosomal proteins and other proteins involved in protein translation were down-regulated at early stages of (4 hpi) IBCDC-RG7 infection as compared to WB-NIV2664. Cell cycle regulator genes such as CDK5 and Cyclin B1 showed up-regulation only at 4 hpi but not at 8, 16 and 24 hpi. Apoptotic genes were significantly down-regulated at 16 hpi and 24 hpi. Genes involved in immune response such as IRF1, IL15R-α, small inducible cytokine subfamily B (Cys-X-Cys), IL1-β, MHC, class1c were down-regulated at 4 hpi and 16 hpi (Figure 2).

**Table 2:** Significantly enriched Gene Ontology terms in response to RG modified H5N1 and highly pathogenic H5N1 infection.

GO Term (Biological processes)	Gene Count	P-value
**RG modified H5N1 (A/India/NIV/2006(H5N1)-PR8-IBCDC-RG7)**		

GO:0042981~regulation of apoptosis	45	4.45E-19

GO:0042127~regulation of cell proliferation	36	1.99E-12

GO:0043066~negative regulation of apoptosis	23	8.59E-11

GO:0043065~positive regulation of apoptosis	25	1.02E-10

GO:0051726~regulation of cell cycle	21	1.09E-09

GO:0006468~protein amino acid phosphorylation	28	7.60E-09

GO:0045859~regulation of protein kinase activity	19	7.55E-08

GO:0019221~cytokine-mediated signaling pathway	10	9.14E-08

GO:0016477~cell migration	16	5.87E-07

GO:0000086~G2/M transition of mitotic cell cycle	6	3.15E-06

GO:0051384~response to glucocorticoid stimulus	8	2.90E-05

GO:0043122~regulation of I-kappaB kinase/NF-kappaB cascade	9	2.96E-05

GO:0034330~cell junction organization	7	4.44E-05

GO:0006260~DNA replication	11	6.29E-05

GO:0046649~lymphocyte activation	11	9.26E-05

GO:0045321~leukocyte activation	12	1.01E-04

**HPAI-H5N1 (A/Chicken/India/WB-NIV2664/2008)**		

GO:0042981~regulation of apoptosis	29	1.35E-13

GO:0042127~regulation of cell proliferation	26	2.83E-11

GO:0043066~negative regulation of apoptosis	17	1.27E-09

GO:0006954~inflammatory response	15	2.81E-08

GO:0045597~positive regulation of cell differentiation	13	3.55E-08

GO:0043065~positive regulation of apoptosis	16	1.38E-07

GO:0001932~regulation of protein amino acid phosphorylation	11	2.14E-07

GO:0006952~defense response	18	5.11E-07

GO:0045321~leukocyte activation	12	5.73E-07

GO:0006979~response to oxidative stress	10	1.38E-06

GO:0010740~positive regulation of protein kinase cascade	10	1.61E-06

GO:0051726~regulation of cell cycle	13	1.87E-06

GO:0030098~lymphocyte differentiation	8	5.38E-06

GO:0046649~lymphocyte activation	10	6.80E-06

GO:0019221~cytokine-mediated signaling pathway	7	6.83E-06

GO:0048534~hemopoietic or lymphoid organ development	11	8.57E-06

GO:0006955~immune response	17	1.10E-05

GO:0030595~leukocyte chemotaxis	5	1.02E-04

GO:0042113~B cell activation	6	1.48E-04

### Comparative analysis of host gene expression responses between HPAI-H5N1 and RG modified H5N1 virus infections

**Figure 3:**
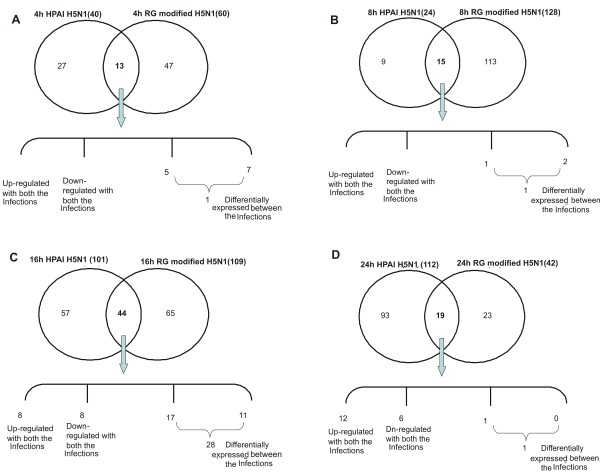
**Comparative analysis of gene expression changes between HPAI-H5N1 (A/Chicken/India/WB-NIV2664/2008) and RG modified H5N1 (A/India/NIV/2006(H5N1)-PR8-IBCDC-RG7) infected A549 cell lines at different post-infected time points**. Venn-diagram showing the common genes between highly-pathogenic and RG modified H5N1 infected A549 cells at **A**. 4 hpi time point **B**. 8 hpi time point **C**. 16 hpi time point **D**. 24 hpi time point.

**Figure 4:**
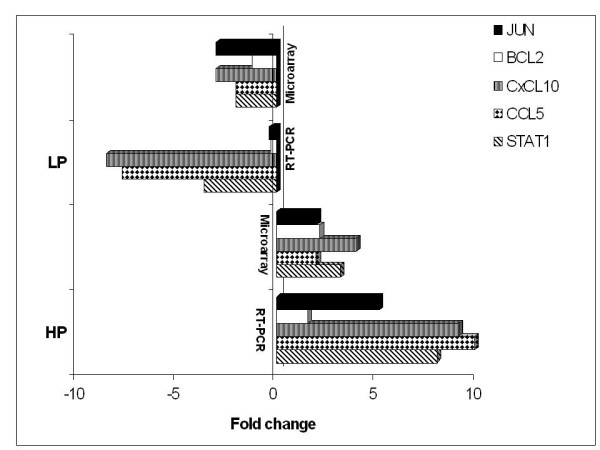
**Validation of microarray data by real time PCR**. Genes showing differential expression between HPAI-H5N1(HP) and RG modified H5N1(LP) virus infections at 16 hpi in A549 were selectively taken for RT-PCR analysis. The expressions of these genes were found to be matching with the microarray analysis.

Gene expression data was compared separately at each post infection time points between the two virus infections (Figure 3). Significantly higher numbers of differentially expressed genes were observed at 16 hpi (Figure 3). A total of 44 genes were found to be common between the two virus infections at 16 hpi. Out of them 8 genes were up-regulated and 8 genes were down-regulated in response to both the virus infections. However, 28 genes were found to have differential expression pattern and were mainly involved in Cytokine-cytokine receptor interaction, Toll like-receptor mediated signaling and p53 signaling pathway [Figure S1 (Additional file 3) and Figure S2 (Additional file 4)]. Cytokines - CXCL10 and RANTES were up-regulated by 4 and 2 folds respectively in WB-NIV2664 infected cells but were down-regulated by 3 and 2 folds respectively in response to infection with IBCDC-RG7 (Table 3). Transcription factors v-JUN and NF-κB were up-regulated in response to WB-NIV2664 infection but were down-regulated in infection with recombinant RG modified H5N1 (Table 3). STAT1, which plays a significant role in JAK-STAT signaling pathway and has been reported to be involved in host immune response to virus infections, was found to be differentially expressing between the two virus infections in our study. Surprisingly, cell cycle regulator Cyclin B1 was down-regulated in WB-NIV2664 infected cells but up-regulated in IBCDC-RG7 infected cells. Pathway analysis using KEGG [Kyoto Encyclopedia of genes and genome http://www.genome.jp/kegg/] tool reveled that these differences in expression profile between cells infected with two virus strains could manifest into differential cell cycle progression and immune response. Expression of selected genes was validated using Real-time PCR, which correlated with the microarray results (Figure 4).

**Table 3:** Genes showing contrasting expression pattern between HPAI-H5N1 and RG modified H5N1 virus infection in A549 cells at 16 hpi.

GENES	HPAI-H5N1 (A/Chicken/India/WB-NIV2664/2008)	RG modified H5N1(A/India/NIV/2006(H5N1)-PR8-IBCDC-RG7)
IL2R-alpha	1.5	-1.5

CXCL10	4.0	-3.0

CCL5(RANTES)	2.0	-2.0

IL1-alpha	2.0	-2.0

IL15R-alpha	2.6	-2.8

JUN	2.0	-2.8

STAT1	3.2	-2.0

FAS	2.0	-2.0

CyclinB1	-2.0	2.0

## Discussion

The present study demonstrates that the host gene expression responses to the highly pathogenic and recombinant H5N1 viruses were qualitatively similar but quantitatively different. The different time points of virus infection or different stages of virus life cycle played an important role in the host gene expression responses. Maximum differences in the host gene expression profile in response to both the virus infections were observed at 16 hpi. This time point is important because at this stage large number of completely assembled virus progeny particles inside the cells give rise to increased host immune responses [17]. Contrasting differences in the expression of various genes between the two infections were found at 16 hpi.

It was interesting to observe significant increase in the expression of many cytokines and transcription factors in response to H5N1 (WB-NIV2664) virus infection but decrease in the expression of same genes in infection with RG modified H5N1 (IBCDC-RG7) at 16 hpi. These cytokines which mainly included IL2, IL1, CXCL10 and RANTES were reported to be involved in cytokine-storm in response to viral infection in humans [18,19] and thus associated with H5N1 virulence.

**Figure 5:**
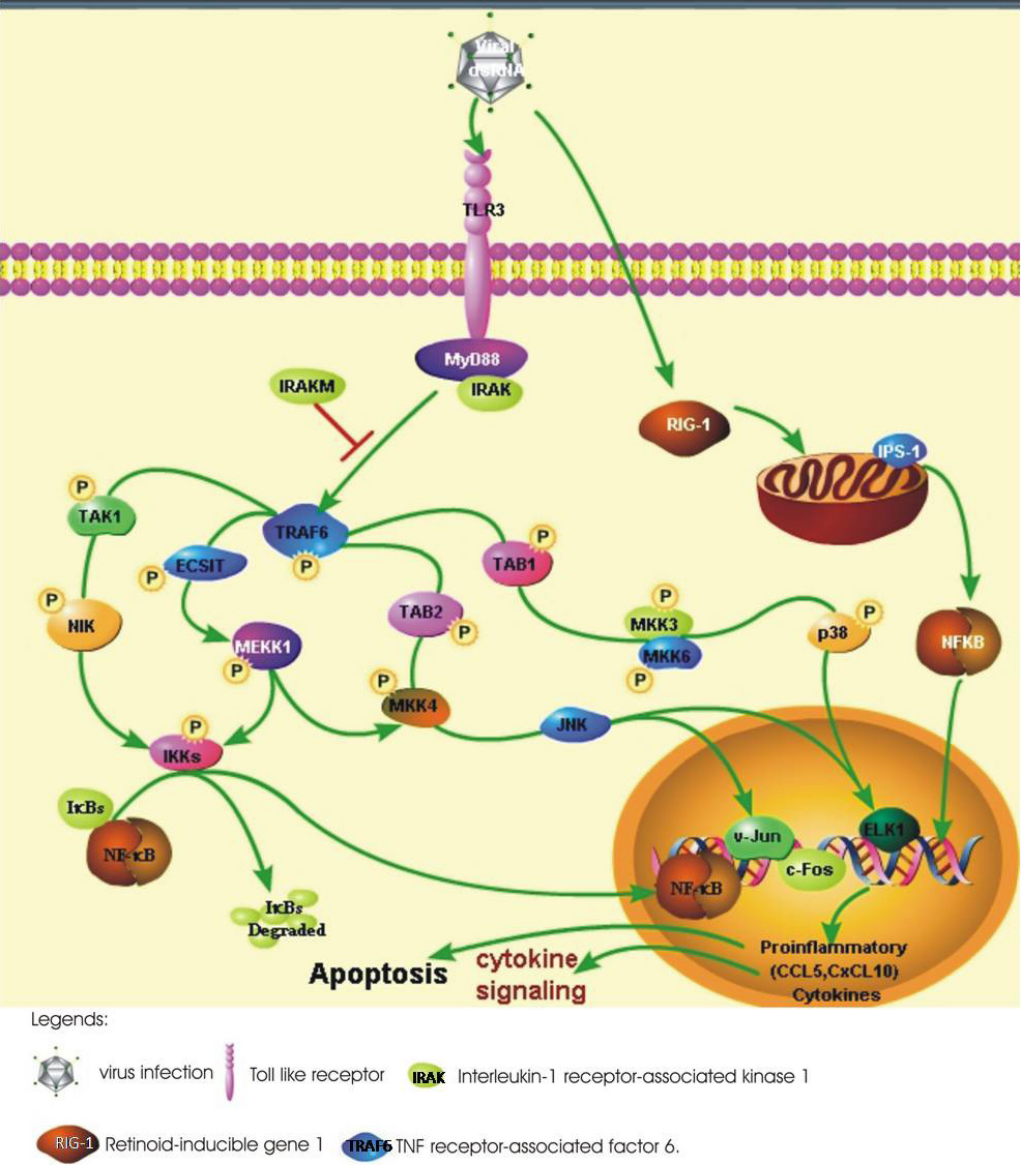
**A model depicting a probable cellular response to HPAI-H5N1 virus infection in A549 cells which are not activated in response to RG modified H5N1 virus infection**. Influenza virus infection results in activation of various signaling events in the host cells. In response to HPAI-H5N1 infection, Toll-like receptor (TLR) mediated signaling events result in activation of inflammatory cytokines like CXCL10, CCL5 through activation of specific transcription factors like NF-κB and v-JUN, as observed in our study. However, this mechanism does not get activated in response to RG modified H5N1 as evident by the down-regulation of cytokine genes. The transcription factors like NF-κB and JUN were also found to be down-regulated during RG modified H5N1 infection.

In spite of overall similar cellular responses in both the infections, WB-NIV2664 was found to be a compelling inducer of cytokines CXCL10 and RANTES than IBCDC-RG-7. This differential expression of cytokines could result in a totally different host cellular response to both the virus infections. A hypothetical model showing probable cellular response to HPAI-H5N1 infection has been shown in figure 5. This type of signaling events might not get activated during RG modified H5N1 infection.

Differential expression of STAT1 in the two virus infections observed in our study also indicate higher cytokine mediated inflammatory responses in WB-NIV2664 infection than the modified strain [20]. IRF-1 has been shown to activate STAT1 and regulates TNF-related apoptosis-inducing ligand (TRAIL) in HIV-1-infected macrophages [21]. The up- regulation of STAT1 in our experiments could be due to IRF1 mediated signaling. Up-regulation of NF-κB and v-JUN observed in this study in response to WB-NIV2664 infection may provide necessary signals required for better virus entry and synthesis of viral proteins inside the cells [22,23].

Cytokine dysregulation plays a major role in pathogenesis of influenza A (H5N1) viruses [5]. Studies on ferrets and nonhuman primates [11,24] as well as on human macrophages [10] have clearly demonstrated the increased cytokine response during H5N1 infection.

The differences in the constitution of the internal genes between the subtypes of influenza viruses may possibly play an important role in differential host gene expression responses. Internal genes of H5N1 viruses, like non structural (NS1) and polymerase basic protein 2 (PB2) have been correlated with host immune responses and high pathogenicity [4,25]. Moreover, it is well established that presence of multiple basic amino acids in the cleavage site of HA is critical for high pathogenicity and systemic spread of H5N1 viruses [4]. Replacement of six internal genes with A/PR/8/34 and absence of multibasic amino acids at the HA cleavage site of IBCDC-RG7, could be a vital reason for its inability to induce host cytokine response similar to WB-NIV2664. Hence, our data supports that these host responses are probably driven by intrinsic differences of gene constitution of the H5N1 viruses.

Up-regulation of apoptotic genes like BAX, BAK1 in H5N1 (WB-NIV2664) but not in IBCDC-RG7 infection could be a part of cytokine mediated response [26]. The higher expression of apoptotic genes could explain higher amount of tissue damage observed in other studies during H5N1 infection. Among various viral factors, NS1 has been reported earlier to induce caspase-dependent apoptosis in human alveolar basal epithelial cells [25]. NS1 protein of H5N1 might have a role in enhancing expression of apoptotic factors leading to high virulence.

## Conclusion

Thus, our findings show that HPAI-H5N1 is a better inducer of inflammation and cytokine mediated apoptosis compared to the RG modified H5N1 at a very specific stage of infection (16 hpi) which could explain its high pathogenicity. This study highlights the role played by the viral factors in inducing host defense mechanism by modulating host gene expression response.

## Abbreviations

HPAI: (highly pathogenic avian influenza virus); hpi: (hours post infection); aRNA: (amino allyl amplified RNA); RG modified: (Reverse genetics modified); GO: (gene ontology)

## Competing interests

The authors declare that they have no competing interests.

## Authors' contributions

AKC and ACM conceived and designed the experiments. AKC, VCV, SM, RS and SDP performed the experiments. AKC, VCV, SM performed data analysis and bioinformatics studies. AKC, SM and ACM wrote the paper. All authors read and approved the final manuscript.

## Supplementary Material

Additional file 1 **Table S1. List of significantly up-regulated and down-regulated genes in A549 cells infected with HPAI-H5N1 at different post-infection time points**. Genes showing increase or decrease in expression by ≥ 1.5 folds (Significant, p-value < 0.05) compared to controls at different post infection time points studied with HPAI-H5N1 have been enlisted. Click here for file

Additional file 2 **Table S2. List of significantly up- and down-regulated genes in A549 cell lines infected with RG modified H5N1 at different post-infection time points**. Genes showing increase or decrease in expression by ≥ 1.5 folds (Significant, p-value < 0.05) compared to controls at different post infection time points studied with RG modified H5N1 have been enlisted. Click here for file

Additional file 3 **Figure S1. Genes involved in chemokine & cytokine mediated signaling in highly-pathogenic and RG modified H5N1 infected A549 cell lines (16 h post infection time point)**. Red arrow indicates expression in highly-pathogenic H5N1 infected A549 cells and Blue arrow indicates expression in RG modified H5N1 infected A549 cells. Up- arrow indicates up-regulation and down-arrow indicates down-regulation. Click here for file

Additional file 4 **Figure S2. Genes involved in p53 signaling pathway in highly-pathogenic and RG modified H5N1 infected A549 cell lines (16 h post infection time point)**. Red arrow indicates expression in highly-pathogenic H5N1 infected A549 cells and Blue arrow indicates expression in RG modified H5N1 infected A549 cells. Up- arrow indicates up-regulation and down-arrow indicates down-regulation. Click here for file
