# Supplementary material for: Host gene expression profiling in influenza A virus-infected lung epithelial (A549) cells: a comparative analysis between highly pathogenic and modified H5N1 viruses
Source: Virol J. 2010 Sep 9;7:219. doi: 10.1186/1743-422X-7-219 (PMC2945955; doi:10.1186/1743-422X-7-219)
Supplement: Additional file 1 — Table S1. List of significantly up-regulated and down-regulated genes in A549 cells infected with HPAI-H5N1 at different post-infection time points. Genes showing increase or decrease in expression by ≥ 1.5 folds (Significant, p-value < 0.05) compared to controls at different post infection time points studied with HPAI-H5N1 have been enlisted. [file 1743-422X-7-219-S1.DOC]

Table S1**. List of significantly up- and down-regulated genes in A549 cell lines infected with HPAI-H5N1 (A/Chicken/India/WB-NIV2664/2008) at different post-infection time points**.

| **Gene ID** | **Description** | **Fold change** |
| --- | --- | --- |
| **4 hpi** | | |
| NM_002963 | S100 calcium-binding protein A7 (psoriasin 1) | 2.1297483 |
| U69127 | far upstream element (FUSE) binding protein 3 | -2.124454 |
| **8 hpi** | | |
| NM_000210 | integrin, alpha 6 | -2.6230335 |
| NM_006206 | platelet-derived growth factor receptor, alpha | -2.6103954 |
| NM_002268 | karyopherin alpha 4 (Qip1) | -2.3417711 |
| **16 hpi** | | |
| NM_004049 | BCL2-related protein A1 | 2.189591 |
| NM_001964 | early growth response 1 | 2.207526 |
| NM_000994 | Ribosomal protein L32 | 2.514103 |
| NM_001657 | amphiregulin (schwannoma-derived growth factor) | 2.578047 |
| NM_001032 | Ribosomal protein S29 | 2.607416 |
| NM_002189 | interleukin 15 receptor, alpha | 2.608685 |
| NM_000268 | neurofibromin 2 (bilateral acoustic neuroma) | 2.6854 |
| NM_002198 | interferon regulatory factor 1 | 3.134424 |
| NM_007315 | signal transducer and activator of transcription 1, 91k | 3.224348 |
| NM_003603 | Arg/Abl-interacting protein ArgBP2 | 3.31811 |
| NM_004048 | beta-2-microglobulin | 3.339335 |
| NM_001565 | small inducible cytokine subfamily B (Cys-X-Cys), | 4.358359 |
| NM_001200 | bone morphogenetic protein 2 | 4.652885 |
| NM_002067 | guanine nucleotide binding protein (G protein), alpha 11 (Gq class) | 4.748868 |
| NM_003390 | wee1+ (S. pombe) homolog | -14.351 |
| NM_003157 | serine/threonine kinase 2 | -7.92899 |
| NM_004536 | baculoviral IAP repeat-containing 1 | -4.70419 |
| NM_005229 | ELK1, member of ETS oncogene family | -4.66394 |
| NM_000221 | ketohexokinase (fructokinase) | -4.3185 |
| NM_002117 | major histocompatibility complex, class I, C | -3.82267 |
| NM_001904 | catenin (cadherin-associated protein), beta 1 (88kD) | -3.60733 |
| NM_003467 | chemokine (C-X-C motif), receptor 4 (fusin) | -2.5603 |
| NM_004354 | cyclin G2 | -2.33702 |
| **24 hpi** | | |
| NM_000584 | interleukin 8 | 2.1922169 |
| NM_001565 | small inducible cytokine subfamily B (Cys-X-Cys), | 2.3936596 |
| NM_000744 | cholinergic receptor, nicotinic, alpha polypeptide 4 | 2.6643968 |
| NM_006180 | neurotrophic tyrosine kinase, receptor, type 2 | -2.06324 |
