# Supplementary material for: Host gene expression profiling in influenza A virus-infected lung epithelial (A549) cells: a comparative analysis between highly pathogenic and modified H5N1 viruses
Source: Virol J. 2010 Sep 9;7:219. doi: 10.1186/1743-422X-7-219 (PMC2945955; doi:10.1186/1743-422X-7-219)
Supplement: Additional file 2 — Table S2. List of significantly up- and down-regulated genes in A549 cell lines infected with RG modified H5N1 at different post-infection time points. Genes showing increase or decrease in expression by ≥ 1.5 folds (Significant, p-value < 0.05) compared to controls at different post infection time points studied with RG modified H5N1 have been enlisted. [file 1743-422X-7-219-S2.DOC]

Table S2**. List of significantly up- and down-regulated genes in A549 cell lines infected with RG modified H5N1 (A/India/NIV/2006(H5N1)-PR8-IBCDC-RG7) virus at different post-infection time points.**

| **Gene ID** | **Description** | **Fold change** |
| --- | --- | --- |
| **4 hpi** | | |
| NM_001565 | small inducible cytokine subfamily B (Cys-X-Cys), | -3.09053 |
| NM_000994 | Ribosomal protein L32 | -2.09476 |
| NM_005180 | murine leukemia viral (bmi-1) oncogene homolog | 2.062517 |
| NM_001338 | coxsackie virus and adenovirus receptor | 2.482498 |
| **8hpi** | | |
| NM_005859 | purine-rich element binding protein A | -5.72255 |
| NM_000629 | interferon (alpha, beta and omega) receptor 1 | -3.98753 |
| NM_000121 | erythropoietin receptor | -3.83773 |
| NM_001338 | coxsackie virus and adenovirus receptor | -3.82074 |
| NM_000210 | integrin, alpha 6 | -2.99664 |
| NM_007294 | breastcancer1,earlyonset | -2.97396 |
| NM_002879 | RAD52 (S. cerevisiae) homolog | -2.86873 |
| NM_002757 | mitogen-activated protein kinase kinase 5 | -2.34318 |
| NM_001527 | histone deacetylase 2 | -2.34189 |
| NM_003286 | topoisomerase (DNA) I | -2.15997 |
| NM_005180 | murine leukemia viral (bmi-1) oncogene homolog | -2.13859 |
| NM_004504 | HIV-1 Rev binding protein | -2.01718 |
| NM_001404 | eukaryotic translation elongation factor 1 gamma | 2.080469 |
| NM_002155 | heat shock 70kD protein 6 (HSP70B`) | 2.083674 |
| NM_001032 | Ribosomal protein S29 | 2.203894 |
| NM_006290 | Tumor necrosis factor, alpha-induced protein 3 | 2.693088 |
| NM_002982 | small inducible cytokine A2 (monocyte chemotactic protein 1, homologous to mouse Sig-je) | 2.70706 |
| NM_003516 | H2A histone family, member C | 2.851324 |
| NM_001565 | small inducible cytokine subfamily B (Cys-X-Cys), | 7.235646 |
| NM_001924 | growth arrest and DNA-damage-inducible, alpha | 9.713105 |
| **16hpi** | | |
| NM_005910 | microtubule-associated protein tau | -5.15303 |
| NM_006293 | TYRO3 protein tyrosine kinase | -4.34585 |
| NM_001792 | cadherin 2, type 1, N-cadherin (neuronal) | -3.43758 |
| NM_001565 | small inducible cytokine subfamily B (Cys-X-Cys), | -3.03696 |
| NM_002189 | interleukin 15 receptor, alpha | -2.85765 |
| NM_002228 | v-jun avian sarcoma virus 17 oncogene homolog | -2.80432 |
| NM_002392 | mouse double minute 2, human homolog of; p53-binding | -2.68366 |
| M15329 | interleukin 1, alpha | -2.37275 |
| NM_002050 | GATA-binding protein 2 | -2.36576 |
| NM_021103 | thymosin, beta 10 | -2.04629 |
| NM_002879 | RAD52 (S. cerevisiae) homolog | 2.005432 |
| NM_002757 | mitogen-activated protein kinase kinase 5 | 2.170122 |
| X68742 | integrin, alpha 1 | 2.226101 |
| NM_002222 | inositol 1,4,5-triphosphate receptor, type 1 | 2.334975 |
