# Supplementary material for: Host gene expression profiling in influenza A virus-infected lung epithelial (A549) cells: a comparative analysis between highly pathogenic and modified H5N1 viruses
Source: Virol J. 2010 Sep 9;7:219. doi: 10.1186/1743-422X-7-219 (PMC2945955; doi:10.1186/1743-422X-7-219)
Supplement: Additional file 3 — Figure S1. Genes involved in chemokine & cytokine mediated signaling in highly-pathogenic and RG modified H5N1 infected A549 cell lines (16 h post infection time point). Red arrow indicates expression in highly-pathogenic H5N1 infected A549 cells and Blue arrow indicates expression in RG modified H5N1 infected A549 cells. Up- arrow indicates up-regulation and down-arrow indicates down-regulation. [file 1743-422X-7-219-S3.PDF]

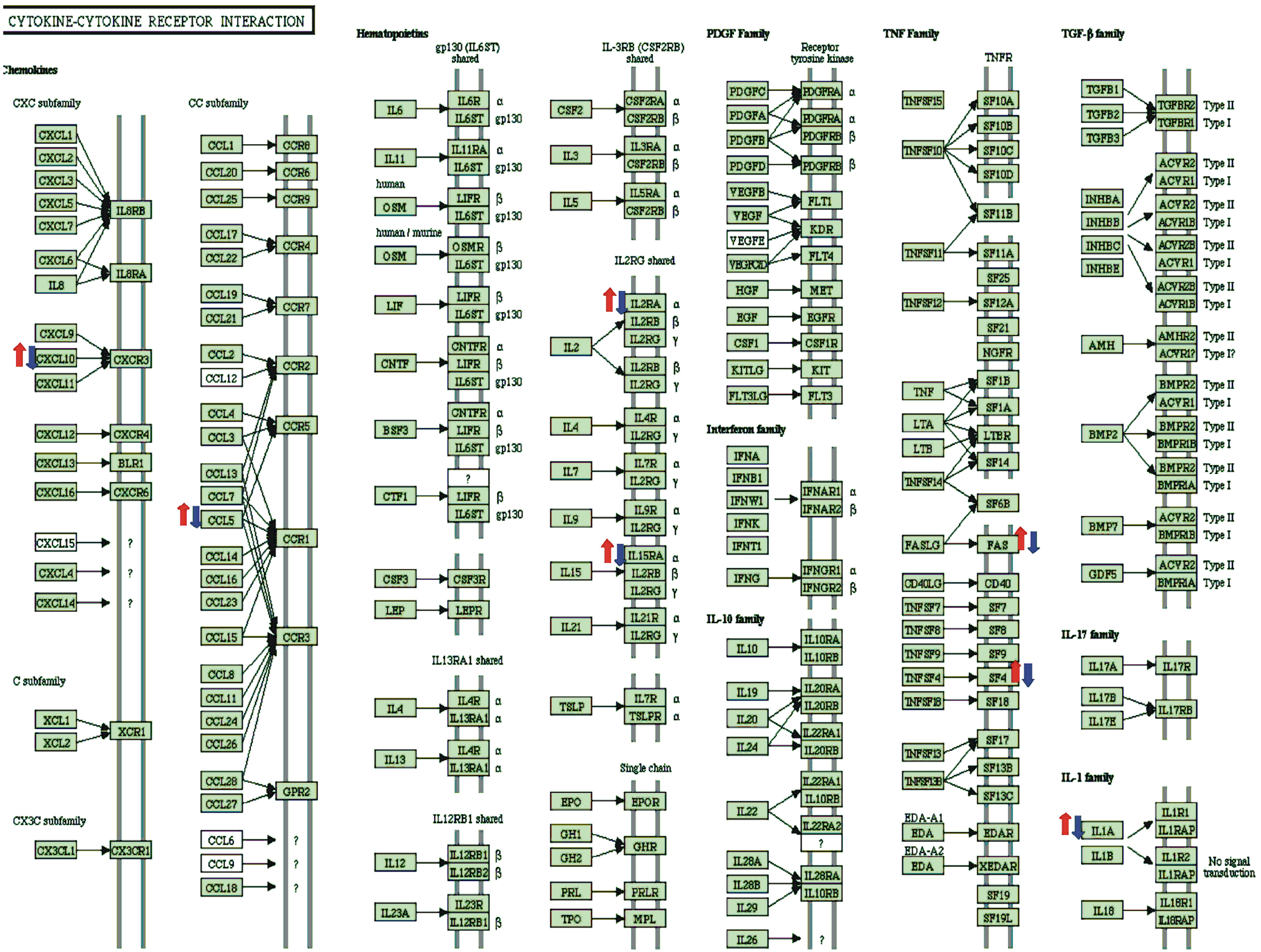

CYTOKINE-CYTOKINE RECEPTOR INTERACTION
Chemokines
CXC subfamily
CXCL1
CXCL2
CXCL3
CXCL5
CXCL7
CXCL6
IL8
IL8RB
IL8RA
CXCL9
CXCL10
CXCL11
CXCR3
CXCL12
CXCR4
CXCL13
BLR1
CXCL16
CXCR6
CXCL15
CXCL4
CXCL14
?
C subfamily
XCL1
XCL2
XCR1
CX3C subfamily
CX3CL1
CX3CR1
CC subfamily
CCL1
CCR8
CCL20
CCR6
CCL25
CCR9
CCL17
CCL22
CCR4
CCL19
CCL21
CCR7
CCL2
CCL12
CCR2
CCL4
CCL3
CCR5
CCL13
CCL7
CCL5
CCR1
CCL14
CCL16
CCL23
CCL15
CCR3
CCL8
CCL11
CCL24
CCL26
CCL28
CCL27
GPR2
CCL6
CCL9
CCL18
Hematopoietins
gp130 (IL6ST) shared
IL6
IL6R α
IL6ST gp130
IL11
IL11RA α
IL6ST gp130
human
OSM
LIFR β
IL6ST gp130
human / murine
OSM
OSMR β
IL6ST gp130
LIF
LIFR β
IL6ST gp130
CNTF
CNTFR α
LIFR β
IL6ST gp130
BSF3
CNTFR α
LIFR β
IL6ST gp130
CTF1
?
LIFR β
IL6ST gp130
CSF3
CSF3R
LEP
LEPR
IL13RA1 shared
IL4
IL4R α
IL13RA1 α
IL13
IL4R α
IL13RA1 α
IL12RB1 shared
IL12
IL12RB1 β
IL12RB2 β
IL23A
IL23R
IL12RB1 β
IL-3RB (CSF2RB) shared
CSF2
CSF2RA α
CSF2RB β
IL3
IL3RA α
CSF2RB β
IL5
IL5RA α
CSF2RB β
IL2RG shared
IL2
IL2RA α
IL2RB β
IL2RG γ
IL2RB β
IL2RG γ
IL4
IL4R α
IL2RG γ
IL7
IL7R α
IL2RG γ
IL9
IL9R α
IL2RG γ
IL15
IL15RA α
IL2RB β
IL2RG γ
IL21
IL21R α
IL2RG γ
TSLP
IL7R α
TSLPR α
Single chain
EPO
EPOR
GH1
GH2
GHR
PRL
PRLR
TPO
MPL
PDGF Family
Receptor tyrosine kinase
PDGFC
PDGFA
PDGFB
PDGFD
PDGFRA α
PDGFRA α
PDGFRB β
PDGFRB β
VEGFB
VEGF
VEGFE
VEGFC/D
FLT1
KDR
FLT4
HGF
MET
EGF
EGFR
CSF1
CSF1R
KITLG
KIT
FLT3LG
FLT3
Interferon family
IFNA
IFNB1
IFNW1
IFNK
IFNT1
IFNAR1 α
IFNAR2 β
IFNG
IFNGR1 α
IFNGR2 β
IL-10 family
IL10
IL10RA
IL10RB
IL19
IL20
IL24
IL20RA
IL20RB
IL22RA1
IL20RB
IL22
IL22RA1
IL10RB
IL22RA2
?
IL28A
IL28B
IL29
IL28RA
IL10RB
IL26
?
TNF Family
TNFR
TNFSF15
TNFSF10
SF10A
SF10B
SF10C
SF10D
SF11B
TNFSF11
SF11A
SF25
TNFSF12
SF12A
SF21
NGFR
TNF
LTA
LTB
SF1B
SF1A
LTBR
SF14
TNFSF14
SF6B
FASLG
FAS
CD40LG
CD40
TNFSF7
SF7
TNFSF8
SF8
TNFSF9
SF9
TNFSF4
SF4
TNFSF18
SF18
TNFSF13
TNFSF13B
SF17
SF13B
SF13C
EDA-A1
EDA
EDAR
EDA-A2
EDA
XEDAR
SF19
SF19L
TGF-β family
TGFB1
TGFB2
TGFB3
TGFBR2 Type II
TGFBR1 Type I
INHBA
INHBB
INHBC
INHBE
ACVR2 Type II
ACVR1 Type I
ACVR2 Type II
ACVR1B Type I
ACVR2B Type II
ACVR1 Type I
ACVR2B Type II
ACVR1B Type I
AMH
AMHR2 Type II
ACVR1? Type I?
BMP2
BMPR2 Type II
ACVR1 Type I
BMPR2 Type II
BMPR1B Type I
BMPR2 Type II
BMPR1A Type I
BMP7
ACVR2 Type II
BMPR1B Type I
GDF5
ACVR2 Type II
BMPR1A Type I
IL-17 family
IL17A
IL17R
IL17B
IL17E
IL17RB
IL-1 family
IL1A
IL1B
IL1R1
IL1RAP
IL1R2
IL1RAP
No signal transduction
IL18
IL18R1
IL18RAP
