# Supplementary figures and images for: Host gene expression profiling in influenza A virus-infected lung epithelial (A549) cells: a comparative analysis between highly pathogenic and modified H5N1 viruses
Source: Virol J. 2010 Sep 9;7:219. doi: 10.1186/1743-422X-7-219 (PMC2945955; doi:10.1186/1743-422X-7-219)

# P53 SIGNALING PATHWAY

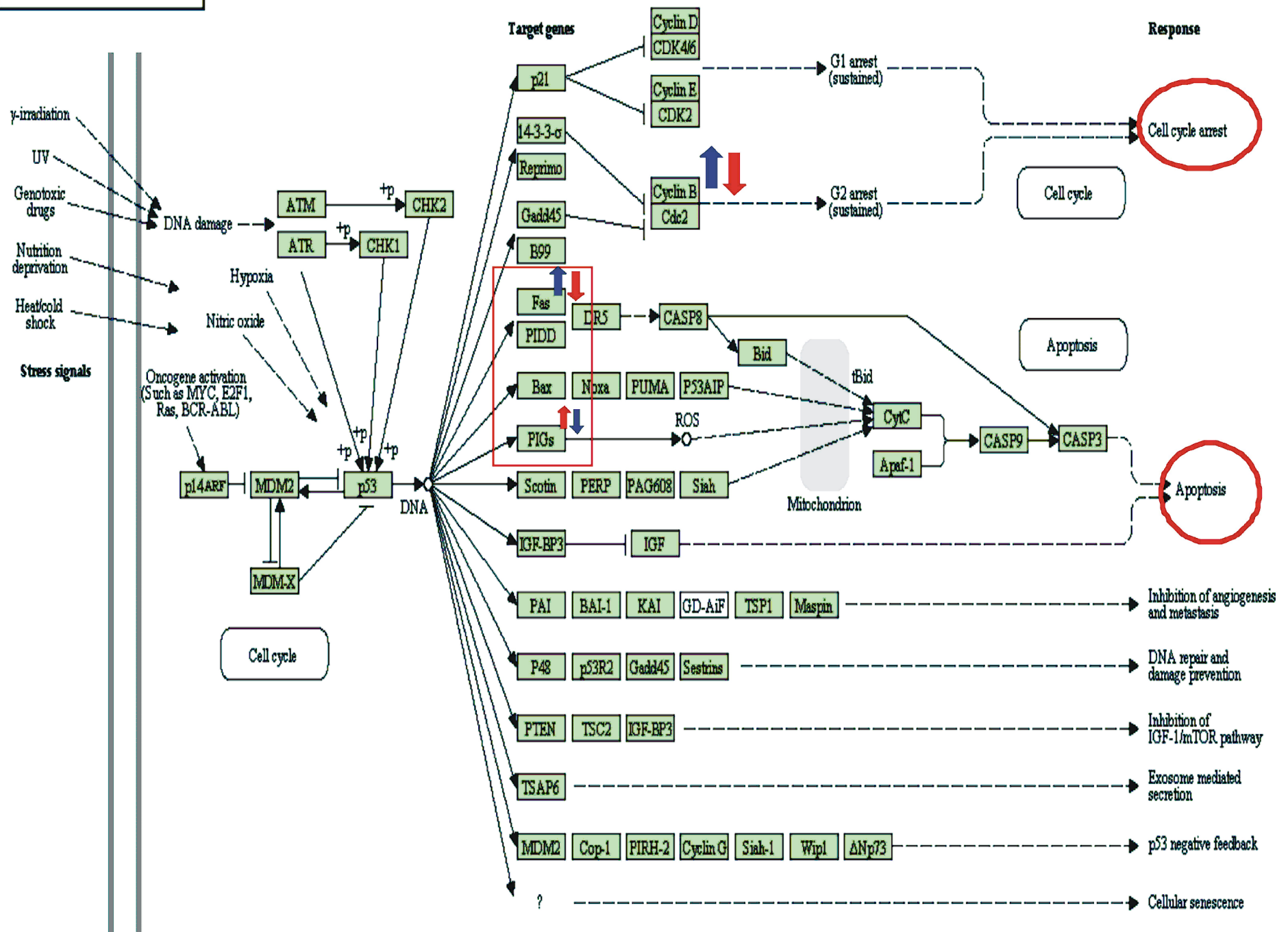

Supplement: Additional file 4 — Figure S2. Genes involved in p53 signaling pathway in highly-pathogenic and RG modified H5N1 infected A549 cell lines (16 h post infection time point). Red arrow indicates expression in highly-pathogenic H5N1 infected A549 cells and Blue arrow indicates expression in RG modified H5N1 infected A549 cells. Up- arrow indicates up-regulation and down-arrow indicates down-regulation. [file 1743-422X-7-219-S4.PDF]
